# Advances of engineered extracellular vesicles-based therapeutics strategy

**DOI:** 10.1080/14686996.2022.2133342

**Published:** 2022-10-20

**Authors:** Hiroaki Komuro, Shakhlo Aminova, Katherine Lauro, Masako Harada

**Affiliations:** a Institute for Quantitative Health Science and Engineering (IQ), Michigan State University, East Lansing, MI, USA; b Department of Biomedical Engineering, Michigan State University, East Lansing, MI, USA

**Keywords:** Extracellular vesicles, engineered extracellular vesicles, drug delivery, EV therapeutics, biomaterials

## Abstract

Extracellular vesicles (EVs) are a heterogeneous population of lipid bilayer membrane-bound vesicles which encapsulate bioactive molecules, such as nucleic acids, proteins, and lipids. They mediate intercellular communication through transporting internally packaged molecules, making them attractive therapeutics carriers. Over the last decades, a significant amount of research has implied the potential of EVs servings as drug delivery vehicles for nuclear acids, proteins, and small molecular drugs. However, several challenges remain unresolved before the clinical application of EV-based therapeutics, including lack of specificity, stability, biodistribution, storage, large-scale manufacturing, and the comprehensive analysis of EV composition. Technical development is essential to overcome these issues and enhance the pre-clinical therapeutic effects. In this review, we summarize the current advancements in EV engineering which demonstrate their therapeutic potential.

## GRAPHICAL ABSTRACT

**Figure:**
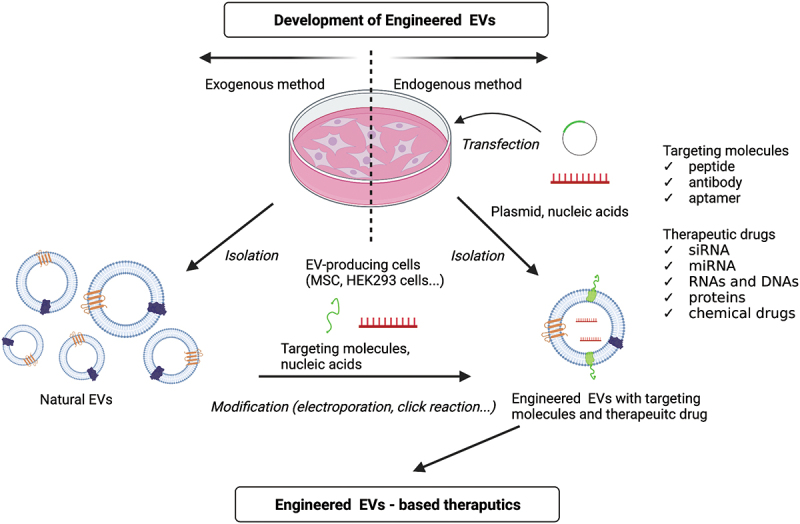

## 1. Introduction

### 1.1. History

The journey of Extracellular vesicle (EV) discovery began in 1946 by Chargaff and West, who found that platelet-free particles exhibit procoagulant properties in plasma [1]. Subsequently in 1967, Peter Wolf first used the term ‘platelet-dust’ for these lipid-rich coagulant particles [2]. In 1969 and 1970, these lipid vesicles were first named after their release from muscle and bone cells as ‘calcifying globules’ and ‘bounded matrix vesicles’, respectively [3,4]. The following year, two research groups used blood cell and plasma models to depict the process of EV release from shearing off the cytoplasmic membrane and budding of internal components of the cell [5,6]. Trams et al. first referred to EVs resulting from this release process as exosomes in the early 1980s [7]. Although the observations of cell-secreted vesicles revealed the lipid bilayer structure and the size as small as 100 nm in 1982, the first report of EV function did not take place till the 1990s [8,9]. Raposo et al. demonstrated that B lymphocyte-derived exosomes activated T-cell responses, similar to major histocompatibility complex (MHC) antigen-presenting complexes [10]. In 2007, Valadi et al. first reported EV-mediated mRNA and microRNA (miRNA) transfer between cells [11]. Since the establishment of the International Society for Extracellular Vesicles (ISEV) in 2011, EV research has continued growing in numbers. In addition, ISEV launched an academic journal called the Journal of Extracellular Vesicles in 2012, which devised guidelines [12] and position papers [13–19]. Our knowledge of EVs and their role in intercellular communication rapidly expanded over the past decade and continue to grow.

### 1.2. EV type

**Figure 1.**
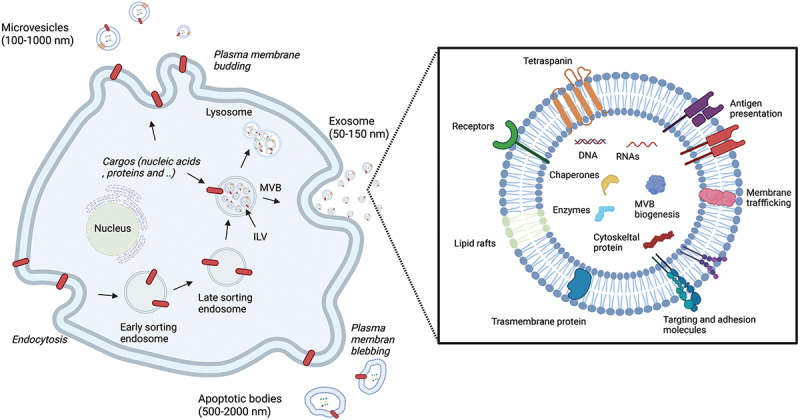
Schematic illustration of EVs biogenesis and composition. Exosomes are formed from the intraluminal budding of MVB and are released into the extracellular space. Microvesicles are produced by budding to the plasma membrane, and apoptotic bodies are produced by budding of the apoptotic cell membrane outward while the cell is undergoing apoptosis. Exosomes carry with a variety of components including nucleic acids, protein, lipids, and metabolites to mediate intercellular communication. MVB; multivesicular bodies, ILV; intraluminal vesicles.

Since all types of cells secrete EVs, their recovery has been reported in all bodily fluids [20], including blood [21], urine [22], saliva [23], breast milk [24], semen [25], bronchoalveolar lavage fluid [26], synovial fluid [27], cerebrospinal fluid [28], and amniotic fluid [29]. EVs are broadly classified into three types: exosomes, microvesicles, and apoptotic bodies (Figure 1). The classification of EV can be associated with their biogenesis. Exosomes (50–150 nm) derive from the inward budding of the endosomal compartment, multivesicular bodies (MVBs). Upon fusion with the cell membrane, exosomes are released to the extracellular space and mediate intercellular communication by transferring bioactive molecules such as RNAs, proteins, and lipids between cells in living organisms [30]. Tetraspanins belong to a cell surface protein family with four transmembrane domains required for intraluminal vesicle formation [31]. CD9, CD63, and CD81 are tetraspanin proteins found enriched in exosomes and often used as EV surface markers [12]. Microvesicles (100–1000 nm), on the other hand, are secreted directly from the plasma membrane. Microvesicles are not as well studied or defined as the other EV types, resulting in more broad characteristics. Apoptotic bodies (100–5000 nm) are produced from apoptotic cells when they undergo programmed cell death. Numerous studies illustrate the heterogeneity of these EVs and missing pieces of information left uncovered, implicating the change in descriptions in the future [12]. For example, there is no defined method or unique maker to separate one EV type from another. A report suggested that 100,000 g ultracentrifugation (UC) co-isolates two EV subtypes defined by specific proteins [32], proposing the importance of considering heterogeneity and diversity among EV types in the study design. Furthermore, some EV subtypes are named after the cell from which they are secreted, such as cardiosome (cardiovascular-origin) and oncosome (oncology-origin) [33–36], though ISEV strongly suggests that the generic term EV be widely adopted [37]. Moreover, the guidelines published by ISEV in 2018 recommend referring to small EVs (sEVs) (<100 nm or <200 nm) or >200 nm as medium/large EVs (m/lEVs) EVs based on their physical characteristic, size and density [12]. This article will adhere to the term ‘EV’ in place of exosome or the names used in the original literature to avoid terminology ambiguity.

### 1.3. Biogenesis

The first observed step begins when endocytosis occurs in the cells. Here, early endosomes are formed followed by late endosomes, which develop into MVBs [38]. Subsequently, a proportion of the MVB fuses with the lysosome membrane, and the intraluminal vesicles (ILVs), which are sEV precursors, and other inclusions are degraded. When the MVB fuses with the plasma membrane, the ILVs in the MVB are released from the cell. Upon release into the extracellular space by exocytosis, these ILVs are subsequently known as sEV [39,40]. Molecules such as ceramide, Hrs, a subunit of the endosomal sorting complexes (ESCRT), and Rab, a small G protein involved in the intracellular trafficking of vesicles, are all involved in this process [41–44]. sEV contain TSG101 and Alix proteins [45], which relate to ILVs formation in ESCRT. Inhibiting the function of these Rab family molecules decreases sEV production [46,47]. Membrane-bound proteins, SNAREs, are also involved in the fusion of MVB with the plasma membrane and the exocytosis [48,49]. Cellular stress and the environment may regulate EV production [47,50–52].

### 1.4. EV function

Functional studies demonstrated that EVs play an important role in intercellular communication by encapsulating nucleic acids, proteins, and lipids that are exchanged in this process [20,53]. Furthermore, EVs have been found to contain mRNAs and miRNAs, which can affect the behavior of the recipient cells [11,30,54]. For example, mouse mast cell-derived EVs transferred to human mast cells, and exosomal mRNA could be translated in protein. This finding prompted the concept that EVs are mediators of intercellular signaling networks composed of diverse cells. Hunter et al. examined EV miRNA expression from the plasma of healthy donors and peripheral blood mononuclear cells [55]. From 420 miRNA expression profiles, they identified an abundant amount of miRNA in both EV samples. Together, they are predicted to be associated with hematopoietic cell homeostasis and metabolism. Additionally, B cells infected with Epstein-Barr virus (EBV) were shown to release EVs containing EBV-associated miRNAs. Thus, the EVs accumulated in non-infected nearby monocyte-derived dendritic cells and miRNAs containing the EVs suppressed the expression of immunostimulatory gene *CXCL11* [56]. EVs are associated with various diseases, such as cardiovascular [57–59], inflammatory [60,61], neurodegenerative [62], cancer [63,64], as well as other diseases [65,66]. For example, B lymphocytes stimulate T cell proliferation and inhibit tumor growth by secreting EVs that display MHC class -I, MHC- II, and T cell costimulatory molecules (CD86) [10,63]. Patients’ cell-derived EVs will have MHC class I/II and will not provoke an immune response. Thus, the specific transfer of bioactive molecules in the EVs makes it an attractive candidate for diagnosis and therapy of disease.

### 1.5. Application

Due to their unique properties, EVs have a wide range of diagnostic and therapeutic applications [14,67]. EVs may serve as drug delivery vehicles by packaging therapeutics molecules, as demonstrated by an initial study that used engineered EVs to deliver exogenous small interfering RNA (siRNA) to induce gene-specific silencing in the mice brain [68–70]. High throughput evaluation techniques revealed biological molecular profiling of EVs [71]. In the early 2010s, the databases ExoCarta (http://www.exocarta.org) [72,73], EVpedia (http://evpedia.info) [74], EV-TRACK (http://evtrack.org) [75], and Vesiclepedia (http://microvesicles.org) [76] were developed to facilitate EV research. These databases contain profiles of EVs isolated from animal species, tissues, and cells, which may elucidate the molecular mechanisms and pathophysiology underlying various diseases. In addition to their therapeutic capabilities, EVs contain predictive biomarkers useful in diagnostics [77–79]. An example, the presence of epidermal growth factor receptor vIII (EGFRvIII), an oncogenic receptor, on microvesicles derived from glioblastoma patient cells that could be exploited for tumor detection [54]. This exemplifies the variety of potential EVs have in clinical models [69]. We continue to learn about EVs at an accelerated pace as we are heading towards diagnostic and therapeutic applications. In particular, some of engineering methods have developed by loading therapeutic drugs and modifying the targeting molecules in EVs for efficient delivery. In this review, we have summarized the latest advances in engineering EVs for these purposes.

## 2. EV engineering method

EVs have inherent nucleic acid and protein cargo delivery properties explored for possible therapeutic use [80]. Among the various cell-derived EVs, Mesenchymal stem cells (MSCs) derived EVs are effective therapeutics due to MSCs homing toward target tissue [81,82]. In addition, induced Pluripotent Stem (iPS)-derived EVs [83] and embryonic stem (ES) cell-derived EVs [83] prevent damage after myocardial infarction; the encapsulated miRNAs in the EVs are associated with these effects. However, naive EVs suffer from a low therapeutic drug loading capacity and a lack of effective delivery capability to the targeting site for clinical application, in addition to their pharmacological mechanisms not being completely understood [49,84,85]. In the past decade, engineered EV has developed surface engineering techniques to deliver vehicles to specific targeted tissues (Section 2.3) and explored effective cargo engineering methods of loading therapeutic agents (Section 2.4) into EVs, thus engineered EVs show promise in new therapeutic development. There are two types of strategies involving EVs surface engineering and cargo loading, the endogenous and exogenous method. In the endogenous method, overexpression of nucleic acid and protein in the producer cells results in the generation of EVs loaded with that cargo or engineered with that surface targeting method before isolation. On the other hand, with the exogenous method, EVs are directly engineered with nucleic acids, therapeutic molecules, and targeting ligands using physical or chemical methods post-isolation. Each of these methods has advantages and disadvantages [86]. In this section, we will discuss the elements involved in the therapeutic use of engineered EVs: EV source (Section 2.1), mass production (Section 2.2), isolation (Section 2.5), storage (Section 2.6), and administration (Section 2.7).

### 2.1. EV source

**Figure 2.**
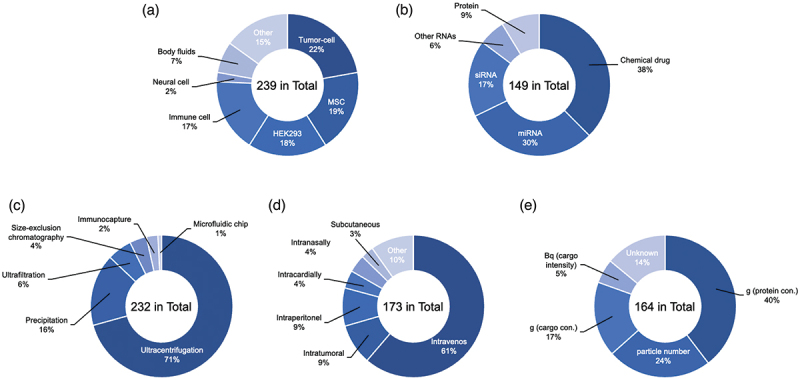
Percentage of publication reports using engineered EVs. (a) Cell-type, (b) Isolation methods, (c) Administration routes, (d) Dose unit, (e) Therapeutic drugs. A systematic literature search search was conducted in the Scopus, PubMed, and Web of Science databases for publications in English through January 2022. The following search terms were used: ‘extracellular vesicle’, ‘exosome’, ‘drug delivery’, ‘therapeutic application’, ‘engineered’, ‘biodistribution’ and related combinations.

EVs can originate from several cell sources, each with their innate differences. Engineered EVs from various origins are used for therapeutic applications (Figure 2(a)). Although EVs from different cellular sources have different biodistribution patterns, which can be exploited for therapeutic purposes [87,88], it is still not discernible which source is appropriate for a specific disease. However, there are sources of EVs that are more prevalently used than others in clinical and research strategy, like MSCs and Human embryonic kidney (HEK) 293 cells.

#### 2.1.1. Mesenchymal stem cell-derived EV

MSCs are multipotent stromal cells that can differentiate into many different cell types [82]. They can be derived from a multitude of tissues, including adipose tissue, liver tissue, bone marrow, umbilical cord blood, placental tissue, amniotic fluid, and dental pulp [89,90]. MSCs are one of the most prevalent cell types in cell therapy due to their role in immunomodulation, tissue regeneration, and treatment of pathological diseases [91]. It has recently proposed that MSCs modulate their therapeutic effects through extracellular factors, therefore, it has hypothesized that EVs are the mediators of the paracrine effects of MSCs [90]. MSCs-derived EVs maintain a lot of the therapeutic properties of MSCs, showing their potential benefits [91]. For this reason, MSCs are currently the most prevalent source of EVs being produced for clinical and research purposes [92].

#### 2.1.2. Immune cell-derived EV

Immune cell-derived EVs can induce either immunostimulatory or immunosuppressive responses [93,94]. EVs isolated from various immune cells, such as dendritic cells (DC), macrophages, natural killer cells (NK), and T-cells, have reported to perform different functions in the body. Therefore, several immune cell-derived EVs are known for having therapeutic potential against immunologic diseases. For example, DC-derived EVs play a key role in adaptive immunity through CD86, CD80, MHC-I, and MHC-II presented on the EV membrane [95]. Also, T-cells-derived EVs exhibit effects of immune activation, and several studies report the inhibition of tumor progression, whereas EVs derived from regulatory T cells (Treg) do not have such functions [96]. NK cells-derived EVs carry NK-cell specific molecules such as FasL and perforin, inducing apoptosis in tumors [97]. Mature DCs-derived EVs have been shown to induce endothelial inflammation and atherosclerosis [98]. Thus, additional knowledge is needed for the selection of immune cells in EV generation for therapeutics.

#### 2.1.3. Tumor cell-derived EV

Tumor cell-derived EVs are an effective source of engineered EVs for cancer treatments. Tumor cell derived-EVs have tumor-specific antigens on their surfaces that stimulate immune cells to trigger an immune response [99]. Tumor-derived EVs showed higher cellular uptake into tumors both *in vitro* and *in vivo* compared to HEK293-derived EVs, which may be affected by tumor-cell type-specific tropism [100]. Furthermore, tumor cell-derived EVs can transfer molecules such as EGFRvIII [101] or mutant K-Ras [102] to neighboring tumor cells to promote escape from the immune system. Since EVs from cancer cells promote tumor progression and metastasis, the effects of these factors must be considered [103].

#### 2.1.4. HEK293-derived EV

HEK293 cell-derived EVs showed minimal toxicologically and immunogenic effects in mice as well as autologous-derived EVs in terms of functionality [104,105]. Some therapeutic agents produced by HEK293 cells have been approved by the US Food and Drug Administration (FDA) or European Medicines Agency (EMA) [106]. To enhance the therapeutic efficacy of EVs from HEK293 cells, cell engineering would be a requirement to load EVs with therapeutic cargo and target them to a specific tissue type. HEK293 cells can easily be manipulated with transfected plasmids or nucleic acids with targeting molecules. In addition, the fast growth and ease of culture of these cells allow for them to be more easily mass produced. EVs derived from several cell sources all exert different biological contents, functions, and biodistribution [107]. Exploiting the unique properties of EVs derived from different cell types will be an important factor for future treatments, including their large-scale generation and drug delivery efficiency. Therefore, in-depth studies are required for the development of EV-based therapies.

### 2.2. Mass production

With their promising clinical applications, there is a demand for creating a standardized method for large-scale EV production. In the laboratory, EV isolation is typically done by UC or filtration techniques. However, even though it varies slightly based on the donor cell type used, the yield of EVs produced from these methods is often too low [108]. Therefore, next-generation approaches are necessary for developing EV mass production techniques to manufacture EVs at an appropriate scale for applicational use. Various cell types have been investigated as possible donor cells. The majority of these EVs are produced from different types of stem cells, epithelial cells, and cancer cells. However, the use of these cell types for large scale EV production comes with a lot of challenges. One of these challenges is that in culture, these cells often grow as adherent monolayers that stop dividing once, they become confluent. This severely limits the number of cells that can be maintained since they require so much space and upkeep. Another challenge is that after a certain number of passages, cell lines build up genotypic and phenotypic changes that lead to senescence, where they eventually stop dividing. This means that new cells need to be constantly obtained from the donor or a multitude of donors, both of which introduce variability [92,106,108]. For example, bone marrow stem cells from older donors proliferate and differentiate slower than those from younger donors [109]. The third challenge is that stem cell lines naturally change and differentiate, which does not warrant a homogenous population of producer cells, thus not producing uniform EVs. This is one of the biggest challenges with MSCs, the biggest source of EVs used for mass production today [92]. Different processing conditions have been investigated to increase the efficiency of EV production. Most of these conditions involve the substitution of culturing cells in *T*-flasks for bioreactors, which are multilayered systems that have mechanisms for performing functions to keep the cells alive [92]. A hollow-fiber bioreactor system resulted in 40-fold the amount of EVs produced per volume of conditioned media in comparison to traditional cell culture techniques [110]. Bioreactors allow for more cell layers to be grown under identical conditions while taking up a smaller amount of space, minimizing manipulation and culture time, and reducing the number of consumables used in traditional culturing methods [111]. However, the bioreactor can also introduce batch variability due to difficulty in monitoring cell conditions. It has also been established that different culturing conditions and stress put on producer cells can influence EV production [92]. Stressors like hypoxia, irradiation, serum starvation, and physical and chemical stress have been known to increase EV production, however, these EVs have the potential to be physiologically different than naturally produced EVs, which is not ideal for the uniformity needed for successful mass production [111]. Due to all these challenges, more work is still needed to standardize EV mass production.

### 2.3. Surface engineering

Although these natural secreted EVs to target specific sites or cells have therapeutic benefits, there are still issues with their targeting ability [49]. Once natural EVs are administered intravenously, they are quickly removed from the bloodstream and mainly accumulate in the lungs, liver, kidneys, and spleen [112–115]. Targeted EV delivery to specific tissues and cells is necessary for their therapeutic use in order to counteract this natural accumulation and is affected by various factors, such as administration route and EV source [107]. To overcome this obstacle, EVs can be modified with targeting molecules to enhance their ability to efficiently travel to the target site. Alvarez et al. showed a method to treat EVs by modifying the EV surface with neuron-specific RVG peptides and loading oligonucleotide siRNA into the EVs for Alzheimer’s disease [70]. Engineering EVs further improves the potential benefits of EV-based therapy. In this section, we will summarize current advancements with EV surface engineering methods for targeted delivery.

#### 2.3.1. Endogenous surface engineering

Endogenous surface engineering adds benefits to EV-based drug delivery. Genetically or metabolically manipulated cells can serve as EV producer cells to add additional functions to EVs, such as targeting capacity and tracking capability.

##### 2.3.1.1. Genetic labeling

Endogenous surface engineering has been a widely used method to target the delivery of EVs to specific sites in the body. Overexpression of a targeting molecule in the producer cell would have difficulty in selectively modifying the molecule on the EV membrane. Alternatively, EV membrane-bound proteins can be used to overcome this issue by presenting the targeting molecule on the surface of EVs [116]. EV membrane-bound proteins used include lysosome-associated membrane glycoprotein 2b (Lamp2b), lactadherin (C1C2), platelet-derived growth factor receptor (PDGFR), -phosphatidylinositol-anchored protein (GPI) and tetraspanin. To produce surface-modified EVs, cells are transfected with plasmid vectors encoding the targeting peptide, antibody, and ligand/glycan, and the secreted EVs are purified. For example, we have shown that engineered EVs prepared from HEK293T cells transfected with the peptide and monobody [117,118]. This method can enhance the targeting ability of EVs by fusing the target protein with the EV transmembrane protein. However, it remains unclear how this modification accurately directs the targeting, and whether the amount of modification can be controlled.

##### 2.3.1.2. Metabolic labeling

Metabolic labeling is another method of exogenous EV surface engineering that is conducted by hijacking cellular biosynthesis processes [119]. Briefly, a sugar derivative containing an azide group or an alkyne group is conjugated to the sugar or precursor to be labeled is taken up by cells. A portion of the target sugar is replaced with the tagged sugar using the metabolic pathway of the cell [120–122]. The principle of bio-orthogonal chemistry is shown to be transferred from cells to EVs [123]. Azide-integrated EVs can be applied to EV imaging by modifying the far-red fluorescent probe Cy3 conjugated dibenzylcyclooctyne (DBCO) [123] or Cy5.5 conjugated DBCO [124] or FITC conjugated DBCO [125] with a click reaction. In addition, this method can be used to modify the EVs membrane with hyaluronic acid, a CD44 targeting molecule, which has been shown to accumulate in CD44-involving immune mouse models, such as tumors and rheumatism [124]. Metabolic engineering can easily functionalize EV surfaces, but it is not clear whether the specificity and efficiency of the surface modification sites can be controlled.

#### 2.3.2. Exogenous surface engineering

The exogenous surface engineering method is used to physically or chemically modify the EV surface with targeting molecules after EV isolation, and there are multiple methods on which this can be achieved. Compared with endogenous techniques, it is easier to alter EVs using this method.

##### 2.3.2.1. Click chemistry

Alternative to the endogenous method, covalent or non-covalent binding of target molecules can also be used to engineer EVs for targeted delivery. Click chemistry, known commonly as covalent binding, is simple and highly efficient EV surface engineering method that works by facilitating the bioconjugation of targeting molecules to EV membranes [126,127]. The reaction is selective between the highly reactive azide and alkyne functional groups, therefore, it can be used for the chemical modification of various biomolecules. In this method, EVs are first modified by grafting an alkyne onto the EV membrane and then reacting it with an azide-linked target molecule via cycloaddition. The surface modification of EVs with the c(RGDyK) peptide has been shown to successfully target EVs to high affinity integrin αvβ_3_ [128], neuropilin-1 targeted peptide (RGE) [129], or fluorophores [130]. However, this alkyne modification method lacks site-specificity, and click chemistry may alter EV surface proteins and structures [131].

##### 2.3.2.2. Hydrophobic insertion

Hydrophobic insertion is a widely used method for liposome-based drug delivery and can be applied to engineering EVs as well [132–136]. This method allows target molecules to functionalize the EV surface using EV membrane components mainly consisting of phospholipids, cholesterol, and glycolipids, without the need for special reactions or reagents. In particular, polyethylene glycol (PEG)-linked phospholipids have been used in a variety of nanomaterials to develop targeted drug delivery [137]. PEG-phospholipids have a hydrophilic tail of PEG and a hydrophobic head of phospholipids, such as distearoyl phosphatidylethanolamine (DSPE) and dimyristoyl-phosphatidylethanolamine (DMPE). The circulation persistence of these PEG-phospholipids can be adjusted by both the length of the PEG chain and the graft density [132,138]. The hydrophobic molecules insertion of the EV membrane is mainly used with in the composition of phospholipid anchor, PEG spacer, and targeting molecules via hydrophobic interaction. For example, it has been reported that DSPE-PEG conjugated to biotin and avidin [139], anisamide (AA) (target sigma receptor) [136], EGa1 (EGFR targeting nanobody) [133], tyrosine-protein kinase met (c-Met) [140], RGD (Arg-Gly-Asp-D-Tyr-Lys monobody, integrin αvβ_3_) [141,142], cyclo-RGD (cRDG) [143], anti-EGFR-iRGD (recombinant protein) [144] on EVs was successful in enhancing targeting. However, the stability of the inserted molecule *in vivo* using this method has yet to be understood [145].

##### 2.3.2.3. Membrane fusion

The lipid bilayer of EVs can easily fuse with liposomes that have similar properties. This fusion method allows properties of the EVs membrane surface to be controlled to increase cellular uptake, colloidal stability, and the half-time in blood. Membrane fusion can be facilitated through a freeze-thaw method, which collapses the liposome-membrane at a lower temperature to promote liposome-EV membrane fusion [146]. PEG-mediated fusion of EVs with fluorescence liposomes enhances the cellular uptake efficiency of hydrophilic compounds [146]. Treatment of EVs with cationic lipids and pH-dependent fusogenic peptides improved cellular uptake and cytosolic release of the cargo [147]. Treatment of cells with azide-conjugated liposome yields EVs with azide groups bound to the membrane. This has been shown to modify tumor-targeting peptides by the click reaction [148]. Exogenous methods can be chosen depending on the properties of the molecule, as various types of EV surface modification methods exist. EV surface engineering should be considered and can be advantageous in enhancing delivery to the targeting site.

### 2.4. Cargo engineering

EVs can be loaded with therapeutic drugs, such as chemotherapeutic agents or nucleic acid molecules for gene therapy. Although there are several studies investigating techniques for loading therapeutic drugs into EVs, some of the methods are still being explored. These developments can be maximized by engineering EVs, and the effect on the EV’s properties should be minimized. Currently, there are two main categories of EV loading methods: endogenous loading (before EV isolation) and exogenous loading (after EV isolation).

#### 2.4.1. Endogenous cargo loading

The first approach, the endogenous method, loads therapeutic drugs into EVs before EVs are isolation from producer cells. In endogenous loading, the encapsulated small molecule acting as cargo in the EV is loaded into vesicles during production by incubating it directly with the parent cells Protein and nucleic acids can also be loaded into EVs by transfection of the producer cell with the encoding DNA. There are various approaches to improving the loading efficiency of nucleic acids into EVs. One approach uses sequence motifs present in miRNAs to control their localization into EVs [149,150]. Certain proteins identified include heterogeneous nuclear ribonucleoprotein A2B1 (hnRNPA2B1) [149], synaptotagmin-binding cytoplasmic RNA-interacting protein (SYNCRIP) [151], Y-box protein 1 (YBX1) [152], which recognize these motifs and bind specifically to exosomal miRNAs and regulate their loading into EVs. Another approach fuses an interaction module with a membrane protein that acts as a marker for EVs and other types of EVs. The method of fusing a protein-binding domain or a sequence-specific nucleic acid-binding domain to tetraspanin (especially CD63, CD9, and CD81), Lamp2b, and a transferrin receptor is used to load any protein or nucleic acid [70,153]. For example, the method of fusing sequence-specific nucleic acid binding motifs such as MS2 [154], L7Ae [155], and Tat [156] to load nucleic acids has been reported. However, these systems are limited in their ability to release the cargo into the cytoplasm in target cells and require further optimization. Examples of this are the iDimerize^TM^ Inducible A/C heterodimer system [157,158], which induces ligand-dependent dimer, and the CRY2-CIB1 system [159], which can control protein interactions with specific wavelengths of light. Collectively, passive incubation in parent cells showed an encapsulation of various therapeutic drugs, which is simple and does not require any special equipment. However, due to the toxicity of EV-producing cells and the inefficient drug loading, there are limitations to its use in applications. Hence, bioinformatic analyses will be further needed to investigate more specific RNA sequencing binding proteins and EV-rich RNA sequencing.

#### 2.4.2. Exogenous cargo loading

The second approach, the exogenous method, involves loading cargo after EV isolation. Exogenous loading methods include co-incubation, electroporation, sonication, saponin, using a transfection reagent, freeze-thaw cycles, and extrusion [160,161]. Although many cases of therapeutic drug loading into EVs have been reported, those methods have multiple parameters that affect loading efficiency and activity, which call for standardization [162]. Co-incubation of EVs with therapeutic drugs is the simplest and most widely used method of exogenous loading. Small hydrophobic molecules such as doxorubicin, paclitaxel, and curcumin can be encapsulated into EVs by co-incubation [115,163]. The loading efficiency of exogenous cargo into EVs depends on the small hydrophobic molecular properties of the cargo. However, small hydrophilic molecules and large molecules such as protein, miRNA, siRNA, and mRNA are difficult to load into EVs using this method. To overcome the limitations of co-incubation, alternative approaches were proposed to improve the membrane permeability of EVs using physical stimulation or chemical drugs. These stimuli are commonly used to improve cellular uptake or induce membrane deformation of cells or liposomes and can be applied to EVs with the same membrane structure. Membrane permeability can be improved by generating pores in the EV membrane, by disrupting membrane integrity, or by transfection with positively charged transfection reagents [164]. One still popular strategy of loading is electroporation [70], which uses electrical pulses to temporarily create a small hole in the EV membrane, allowing drugs to permeate into the EV. However, poor loading efficiency has been reported in some cases, which may result from the formation of siRNA aggregates in the process of electroporation [165]. Sonication is another method used for loading therapeutic drugs into EVs. Sonication uses ultrasound technology to physically disrupt EV membranes and facilitate exogenous drug penetration. An alternative to these methods is saponin, a surfactant molecule that can form complexes with cholesterol in the cell membrane and generate pores, thus increasing membrane permeability. A study has shown that incubation of a small hydrophilic molecule with saponin increases drug loading. However, there are some limitations, such as the concentration of saponin used, due to the need for complete removal of saponin from EVs post-cargo loading due to its hemolytic effects [166]. There are few transfection agents that have been used to load directly therapeutic drugs into EVs and it is still unclear what impact the leftover reagents may cause. The two transfection agents that are currently used are Lipofectamine 2000^TM^ and Exo-Fect^TM^ [167–169]. As an approach to avoid membrane damage, a hybrid loading method that fuses liposomes with EVs was reported. Because of the generally small size of EVs, it is difficult to encapsulate large nucleic acids into them. Most of the current reports on EVs as drug delivery carriers are related to small nucleic acids such as miRNAs and siRNAs and small molecule drugs (Figure 2B). Using this liposome-EV hybrid method, it is easy to encapsulate larger-sized cargo, such as the CRISPR/Cas9 system EVs [170]. Overall, compared to endogenous methods, this method involves various techniques and variable factors in EV cargo engineering methods. The changes in membrane structure and therapeutic molecules due to the physical and chemical stress applied to allow the EVs to take up cargo should be considered. Both approaches require a further search for optimal conditions and exploration of large-scale applications.

### 2.5. Isolation

EVs can be isolated from any bodily fluid in a multitude of ways, each with their own pros and cons in terms of yield and purity [171,172]. The isolation of EVs does not call for one set method as there is research still being done on each of them [12]. The most common and universally used EV isolation method is differential UC (Figure 2C), which utilizes the density of the different particles to isolate EVs [90,171,172]. Though UC can differentiate different subtypes of EVs, it is a relatively high cost in terms of equipment, requires large amounts of starting volume, results in a low yield of EVs, and possibly causes damage to the EVs produced [172,173]. Alternative methods commonly used include size exclusion chromatography (SEC), which uses a porous polymer to separate particles by size [174,175]. SEC is fast and simple while maintaining EV integrity, but there is a significant chance of contamination by similar sized particles, such as lipoproteins [174–177]. Downsides of this method include sheering of EVs due to the pressure placed upon them and clogging of the membrane by larger particles. Another classic and widely used method of isolating EVs is ultrafiltration, a size-based separation of particles. Using this method, the sample is passed through a membrane filter with EV-sized pores, resulting in larger particles not being able to pass through [178,179]. This process is simple, cheap, and allows both small and large sample volumes to be used. Problems of this method are similar to SEC in that it involves clogging of the membrane by the larger particles not being able to pass, resulting in a lower recovery rate, low purity due to similarly sized particles being able to pass, and possible damage to the EVs due to the pressure placed upon them during the isolation process, and contamination with similarly sized particles [179]. Some emerging methods of EV isolation involve microfluidic-based methods, which utilize low sample consumption, analysis time, and ease of use resulting in a high recovery rate and purity, making it ideal for clinical uses [179]. One such technique comes in the form of tangential flow filtration (TFF), a gentler and more scalable option for EV isolation. TFF works similarly to other filtration methods with the addition of a constant circulation of the media that contains the EVs to prevent clogging of the filter by the larger particles. TFF requires less pressure to filter out EVs from the solution, resulting in less damage to them [180,181]. In SEC, contamination of similar sized particles can still occur. In contrast, TFF concentrates the EVs, rather than diluting them in SEC, making them a better fit for large scale isolation [182]. Another emerging method is inertial lift force, which is a passive isolating technique. This method involves lateral migration of particles that focus on distinct microchannels depending on the inertial force experienced by the particle [183,184]. Though it is not yet possible to differentiate EV subtypes, progress is being made. As with any other type of experiment, there is always room for contamination to occur. The most common forms of contaminate found in EV isolation come from the serum that the EVs are being isolated from. As mentioned, there are generally two forms of EV isolation, density gradient-based isolation, UC, and size-based filtration, SEC and TFF. If a size-based isolation method is used, similar sized contaminates can remain with the EVs, for example, lipoproteins such as chylomicrons, very-low-density lipoprotein (VLDL), and VLDL remnants [185,186]. In contrast, if a density-based method is used, then contaminants with similar densities as EVs will remain, such as high-density lipoprotein (HDL) [186,187]. These contaminations can be reduced using two different methods of isolation in succession [176,188]. While some methods are more commonly used than others, currently, there is no standardized method for EV isolation. Each method has its own pros, cons, and situations where one is superior to another, as listed in Table 1.

**Table 1.** Comparison of EV isolation methods.

Isolation method		Advantage	Disadvantage
Ultracentrifugation (UC)-based method	Differential UC	Commonly method, easy operation, long life span, large sample capacity	High cost equipment, time consuming process, protein and lipoprotein contamination risks, low recovery, low purity, morphology changes, low yield
	Density gradient UC	High purity of specific EV populations, easy operation, long life span, high purity, efficient at preserving EV characteristics (suitable analysis)	High cost equipment, time consuming process, low recovery
Size-based method	Ultrafiltration	Time efficient, easy operation, low cost equipment and materials, high EV yield, large sample capacity, higher purity	Protein and lipoprotein contamination risks, EV deformation due to shear stress, loss of EVs due to membrane attachment, low purity, clogging problems in nano-membrane, limited filter lifetime
	Size-exclusion chromatography	Time efficient, easy operation, low cost equipment, high yield, large sample capacity, gravity flow preserves the integrity, and biological activity, removes high density lipoprotein contamination	Contamination with EV-sized nano-particles, low recovery, low purity,
Immunoaffinity-based method	Immunocapture method	Low time consuming process, easy operation, high purity, required less sample volume to isolate, superior RNA yield, high recovery, specific interaction to target EV-specific markers	High cost reagents, unable to be used for large scale production, low yield, difficult to remove from impacted beads, need pre-purification steps
Other techniques	Precipitation	Easy operation, does not require specialized equipment, high yield, preserved EV integrity, large sample capacity, precipitated different type of EVs	High cost reagents, low purity, high background non-EV contamination
	Microfluidic system-based method	Time efficient, small reagent and sample volume used, high affinity, high purity, high sensitivity, analysis can be integrated	Unable to be used for large scale production, lack of global protocols and standardization, requires high technical expertise, low yield, not readily commercially available for chip, specific design required

### 2.6. Storage

Storage is a limiting factor that can impact the integrity and quantity of EVs used for biomedical research and clinical applications [14]. It is recommended that EVs be stored in siliconized containers after being resuspended in phosphate-buffer saline (PBS) [189]. The most common EV storage temperature is −80°C, however, this storage method has limitations when it comes to cost and transportation of samples [190]. In a study by Lőrincz et al [191], it was determined that other storage temperatures might be functional for short-term EV storage. After one day, the number and functionality of EVs stored at −20°C or 4°C dramatically decreased. Storage of EVs at −20°C on the other hand did not have any impact on EV number in the first 28 days. However, EV size and functionality were compromised by day 28. While it has yet to be determined if −20°C is a viable storage temperature for short-term storage of EVs, it serves as a potential option that has reduced cost and transportation issues that arise with the standard −80°C storage temperature. Various freezing methods have also been investigated to try to improve the stability and long-term viability of EVs. Two of these methods are snap freezing and the use of cryoprotectants [192]. However, it was determined that cryoprotectants induced EV lysis and snap freezing did not have any effect on improving the loss function of EV compared to traditional storage methods [191]. A controversy within EV research is the question of if freeze-thaw cycles damage EVs. While some studies report that they do, others say that EVs remain stable after several freeze-thaw cycles [92]. The latest 2022 report on the effects of −80°C storage and freeze-thaw cycles on EV indicates that EV-fusion may have a significant impact on EV functionality. However, no storage conditions were found that would reduce these effects [193]. Moreover, Görgens et al. developed an optimal preservation solution for EV storage and handling [194]. PBS-human albumin and trehalose (HAT) buffer can be used to store EVs at −80°C for up to 2 years and to keep them stable through freeze-thaw cycles. This was shown through several evaluations where PBS-HAT improved the functionality engineered EVs containing green fluorescent protein(GFP)-CD63 intravehicular fluorescence protein and tumor necrosis factor receptor 1 (TNFR1) surface protein compared to other buffers. Furthermore, Wang et al. developed inhaled virus-like particle (VLP) vaccines by modifying the EV surface of lung spheroid cell-derived EV with a recombinant severe acute respiratory syndrome coronavirus 2 (SARS-Cov-2) receptor-binding domain (RBD). They reported the vaccine effectively targets the lungs and can be self-administered by inhalation following storage at room temperature for three months without affecting size, concentration, or RBD levels [195]. A lot is known about EV storage conditions overall, and there is a need for more research on them so a more efficient, standardized storage method can be developed.

### 2.7. Administration route and dose

The choice of administration route and dose of purified engineered EVs is a key factor in achieving future clinical studies. Several doses and routes of administration are being tested, with positive results for distinct types of diseases. The following sections summarize their use with engineered EVs.

#### 2.7.1. Administration route

The method of administration is a crucial factor in elucidating the biodistribution of drug or gene delivery [196,197]. Therapeutic drugs within engineered EVs can be achieved in animal experiments using several administration routes, including intravenous, intratumoral, intraperitoneal, intramyocardial, intranasal, and oral route delivery (Figure 2(d)), and these can be used for the treatment of various diseases. Intravenous administration is the most common method of administration and is widely used in many engineered EV studies. The administered EVs are usually cleared by macrophages and accumulate in the liver, kidney, lung, and spleen [197]. To avoid this issue and to target delivery, disease-specific administration is applied. Intranasal administration has been proposed to bypass the blood-brain barrier (BBB) as a method of administration to the brain [198]. For example, encapsulated curcumin or a signal transducer and activator of transcription 3 (Stat3) inhibitor loaded into EVs were delivered to microglia cells via an intranasal route. The engineered EVs showed significantly delayed brain tumor growth in the tumor model [163]. The intravenous route poses an obstacle due to long circulation time, which can affect the percentage of therapeutic drug encapsulated EVs that have access to the targeted tissue. On the other hand, organ-specific routes such as intramyocardial or intratumoral administration bypass the bloodstream and enable direct injection of drugs directly to the treatment site, achieving high concentrations at target cells and reducing dose requirements. It also counteracts drug uptake by nontarget cells, minimizing systemic toxicity [199,200]. Although those routes can affect EV biodistribution, there are few studies that report biodistribution using multiple administration routes in the same experimental system [107,201].

#### 2.7.2. Dose

The dosing and quantification of engineered EVs vary widely among studies. Gupta et al. investigated the effective dosage in the target disease and observed inconsistency [202]. In preclinical and clinical studies, it is necessary to quantify the amount of engineered EVs to be administered. Currently, there are various methods, such as protein concentration, particle number, and drug concentration equivalent (Figure 2E). These methods have their own advantages and disadvantages. For example, the amount of protein can be easily assessed but may vary depending on the EV source and EV isolation method [203]. The particle number is counted using special equipment such as nanoparticle tracking analysis (NTA). The equipment used may also affect the concentration of the particles and the setting of the measurement conditions, as particle measurement accuracy depends on the sample and the preparation method. There needs to be standardization in the description of these doses.

## 3. EV engineering for imaging and *in vivo* tracking

**Figure 3.**
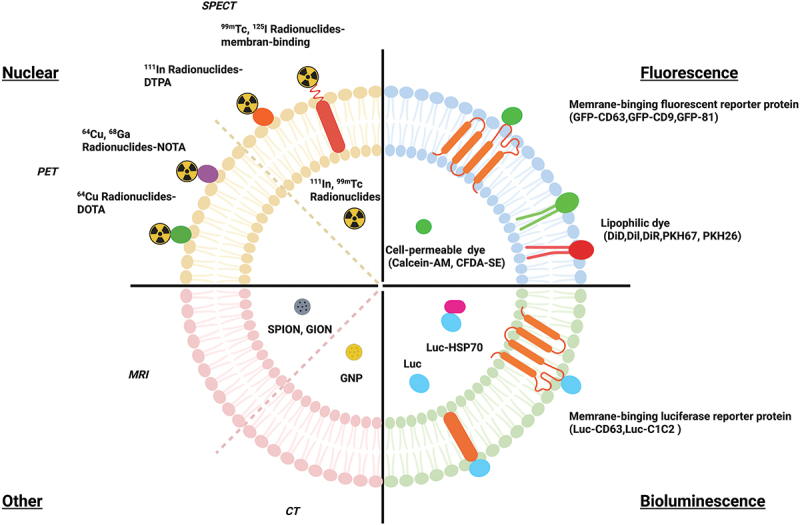
Schematic representation of the four types of engineered EV imaging.

*In vivo* imaging and tracking of EVs is a helpful and useful step in determining the efficiency of targeted delivery, their complex role within processes, and their uptake and half-life [204]. Several challenges remain for tracking EVs *in vivo*, including their small size, rapid dispersion in body fluids, and the lack of high-contrast imaging techniques [205]. For imaging purposes, these EVs can be labeled both exogenously and endogenously. There are generally three types of imaging used for EV studies, including fluorescence, bioluminescence, and nuclear, as shown in Table 2 and Figure 3.

**Table 2.** Summary of engineered EV for imaging modalities.

Imaging modality		Source of detection	Spatial resolution [206]	Sensitivity (M) [206]	Imaging time	Labeling agent	Clinical translation
Fluorescence		Visible light	2–5 μm	10^−9^–10^−12^	Seconds to minutes	Reporter protein (GFP [107,124,147,207–209], RFP [210], mCherry [117])	No
		Visible and infrared light			Seconds to minutes	Lipophilic dye (PKH26/67 [211], Dil,DiD [212], DiR [213], CFDE-SE [214], Calcein-AM [215])	No
Bioluminescence		Visible and infrared light	2–5 μm	10^−9^–10^−12^	Minutes	Luciferase (gLuc [216], Rluc [217], NanoLuc [218], ThermoLuc [218], Luc [219], effluc [97])	No
Nuclear	SPECT	Single γ-rays	4–6 mm	10^−10^–10^−11^	Minutes	Radionucleotide (99mTc-tricarbonyl [220], 99mTc-HMPAO [221], 99mTC [222], 125I-biotin [223], 131I-Na [224], 111In-DTPA)	Yes
	PET	Pairs of γ-rays	2–4 mm	10^−11^–10^−12^	Seconds to minutes	Radionucleotide (64Cu-NOTA [225], 64Cu-DOTA, 68Gd-NOTA, 124I-Na [226])	Yes
Other	MRI	Radio frequency wave	25–100 μm	10^−3^–10^−5^	Minutes to hours	SPIONS [129,227–230], GION [231], GNP [232], DSPE-DOTA-Gd [233]	Yes
	CT	X-rays	50–200 μm	-	Minutes	GNP [234,235], SPION [236]	Yes

### 3.1. Fluorescence imaging

Fluorescence imaging is one of the most popular and easy to manipulate techniques in molecular and cellular biology. Thus, it has become a powerful tool for observing interactions between EVs and cells, as well as cell behavior [237,238]. Fluorescent labeling includes the use of lipophilic dyes such as PKH67/26 [211], Dil [212], DiR [204,218], and DiD [212] or cell permeable dye such as Calcein-AM [215] and CFDA-SE [214] that work by staining the membranes of EVs [204,218]. Other forms of fluorescent labeling include near-infrared (NIR) dyes such as indocarbocyanine analogs, and fluorescent proteins, such as GFP and red fluorescent protein (RFP) [218]. These dyes are generally used in *in vitro* studies as they have limited tissue penetration and tissue autofluorescence [239]. Another disadvantage with this method of labeling is the high risk of the dyes transferring to other cell membranes, making biodistribution studies inaccurate [240–242]. Additionally, these dyes are highly stable and resistant to degradation, making them not ideal for half-life studies, as in certain cases, they can out survive the EVs [218,240]. Advantages of fluorescence labeling include its ease of use that allows for real time tracking of EVs. NIR dyes in contrast to lipophilic dyes are a better fit for *in vivo* experiments, as they have high tissue penetration and low autofluorescence, but they are still not comparable to other non-fluorescence imaging methods, requiring *ex vivo* studies to confirm results [243–245]. Another *in vivo* option is fluorescent proteins which can be combined with EV markers. Some examples of these fluorescent proteins are CD63-GFP [207,246], CD9-GFP [207], and CD81-GFP [208]. These can be produced endogenously by transfecting cells with a CD9-GFP plasmid and isolating the produced EVs that contain that plasmid [207]. By creating a cell type-specific EV reporter (CD63-GFP) mouse model, Men et al. showed that EVs from neurons containing individual secreted miRNAs mediate signaling to glia [246]. Higher production of EVs with these markers results in higher fluorescence, thus making it a possible imaging option *in vivo* [107,247]. A downside of using this method is that the labeling may be restricted to certain subtypes of EVs rather than a wider range [248]. In conclusion, due to these disadvantages that come with using these dyes *in vivo* and the additional verification steps needed to confirm the results, these dyes are generally used for *in vitro* experiments, and other imaging tools are used *in vivo* [245].

### 3.2. Bioluminescence imaging

Bioluminescence is a popular and widely used tool for both endogenous and exogenous labeling for EV imaging and tracking. Bioluminescence involves light emitted from the natural enzyme substrate reaction, unlike the excitation and emission required for fluorescence [155]. Reporters engineered to the EV surface that emit bioluminescence involve gaussian Luciferase (gLuc), NanoLuc, ThermoLuc, and Firefly [218]. Advantages to using this method include not having the problems of fluorescence in terms of autofluorescence, short shelf life, and high expense as seen in radiolabeling [249]. There are many types of luciferases, but they are not all equal, each coming with their own pros and cons. Gupta et al. tested out different reporters both *in vitro* and *in vivo* to see which gave the best brightness and stability in terms of half-life and pH sensitivity [218]. For *in vitro* experiments, nanoLuc performed better than the other reporters, but for *in vivo*, thermoLuc gave a high emission wavelength with no substrate toxicity [218]. A downside is that these bioluminescence reporter proteins require some genetic modifications to the EV or parent cell. One way to do this is to engineer the reporter to create fusion proteins with EV surface markers like lactadherin, which results in the presentation of the reporter protein on the surface of EVs, allowing for them to be visualized [216]. Bonsergent et al. used NanoLuc engineered EV markers *in vitro* that were presented both inside and outside of the EV, using Hsp70 and CD63 respectfully [250]. Additionally, Lai et al. created a metamodel imaging reporter for both fluorescent and bioluminescent *in vivo* imaging that was presented on the surface of EVs [248,251]. To control the expression of the fluorescent and bioluminescent, the researchers fused a biotin acceptor domain to gLuc. In the presence of biotin ligase, the bioluminescent signal was emitted, and with the addition of a biotin binding protein fused to a fluorescent label, fluorescence imaging could take place after injection [251]. Bioluminescence labeling has a multitude of options, each with scenarios where they best fit, that are widely used for *in vivo* imaging.

### 3.3. Nuclear imaging

A relatively newer *in vivo* imaging technique for EVs is radiolabeling. In radiolabeling, radionuclides such as ^125^I-labeled biotin [223], ^99m^Tc-tricarbonyl [220,252], ^125^I-Na [220,252], and ^131^I-Na [224] are used and emit radioactive signals, allowing for live tracking using single photon emission computed tomography (SPECT) or positron emission tomography (PET). These are imaging techniques used in both clinical practice and biomedical research that can assist in visualizing the distribution of administered radioactive substances *in vivo*. They can also provide information about biological functions rather than just anatomical structures [253,254]. Radiolabeling provides excellent tissue penetration and allows for both quantitative and qualitative measurements of EV biodistribution [206]. Radioisotopes also have a longer half-life than EVs allowing for long term EV tracking, though they will remain in the system even after the degradation of the EVs. Some cons of this method include the radioactivity of these isotopes and the minimal number of protocols available currently [255]. Generally, this method of imaging is time consuming and expensive, but more studies are producing faster and more cost-effective methods of using these isotopes for radiolabeling of EVs. Faruqu et al. looked at two methods of radiolabeling EVs, membrane and intraluminal labeling of EVs, both of which did not require EV engineering. They found that membrane labeling was more reliable and provided better imaging than intraluminal labeling [245]. Royo et al. studied if a modification to the surface glycosylation of EVs changed the biodistribution patterns *in vivo* using ^124^I-Na radiolabeled EVs with PET imaging [226]. They found that the modification did change the biodistribution, and PET allowed for accurate quantification and visualization of the process [226]. These radionuclides can be engineered onto the EV surface along with therapeutics and targeting ligands. Morishita et al. engineered EVs endogenously to present a membrane type-1 matrix metalloproteinase (MT1-MMP) specific antibody with ^125^I-IBB using a plasmid vector that was transfected to the parent cells [223]. In conclusion, radiolabeling allows for imaging of deep tissue and quantification of EV biodistribution.

### 3.4. Other imaging

Besides the three current most common methods of *in vivo* imaging, there are always new types of imaging being created. One such method involves using superparamagnetic iron oxide nanoparticles (SPIONs) in combination with magnetic resonance imaging (MRI) or magnetic particle imaging (MPI) [256,257]. SPIONs are magnetic nanoparticles that are commonly used for imaging cells with MRIs in the blood, perfusion, and metastatic tumors, and have recently transitioned to being used to image EVs. Advantages to using MRI over nuclear imaging include the absence of radiation and low toxicity, thus increasing safety and easing the transition to clinical applications. Additionally, SPIONs do not require genetic modification as they can be applied by incubating donor cells with SPION containing media, washing/replacing media, and collecting supernatant after incubation [227], or by electroporation with isolated EVs [228]. The downsides of using SPIONs include possible false positives, low sensitivity, and lack of accurate quantification, unlike nuclear imaging. This can be seen in Jia et al., who engineered EVs to present RGE, a targeting ligand for glioma, and loaded the EVs with Curcumin as a tumor therapeutic and SPIONs for MRI. The researchers found the labeled EVs targeted and had an inhibitory effect on the tumor, while the SPION allowed for visualization of the tumor, leading to possible diagnosis and evaluation uses [129]. Another alternative is gold nanoparticles (GNPs), which are commonly used in combination with computed tomography (CT) imaging and surface Raman spectroscopy (SERS) [234,255]. An advantage that comes with using GNPs is their high biocompatibility and stability [255,258]. Lara et al. labeled B16F10 derived EVs with GNP, PEG, and folic acid (FA) conjugate to assist with the internalization of GNP while studying the biodistribution of the EVs without changing the EV surface. The GNP labeling allowed for precise localization and quantification of the EVs [234]. SPIONS and GNPs are just a couple of up-and-coming alternative EV imaging tools, and depending on the requirement on the experiment, may be a better fit compared to the usual three tools used currently. Collectively, to visualize the biodistribution of EVs, it is possible to track engineered EVs with various imaging modalities. These modalities using engineered EVs would allow for the creation of novel designated EVs for therapeutic applications.

## 4. Surface engineering for targeting specificity

**Figure 4.**
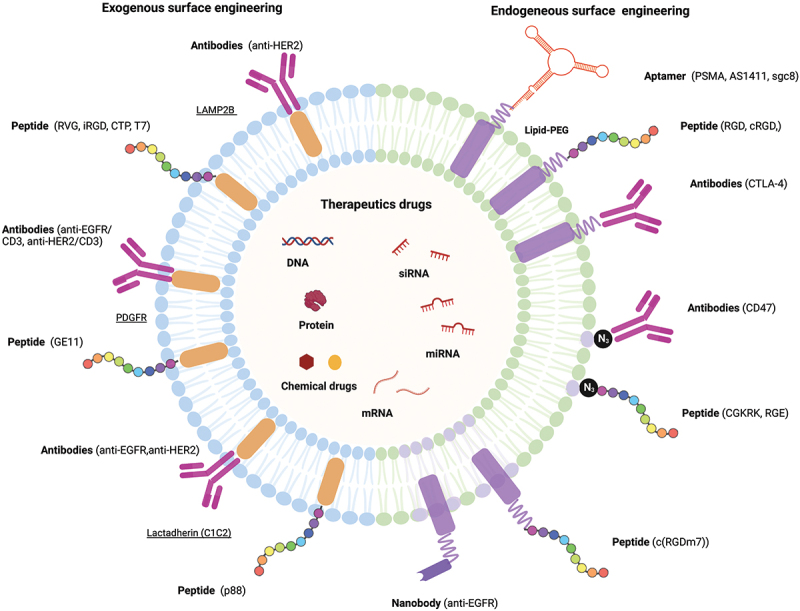
Schematic representation of surface engineering of targeting molecules and loading of therapeutic drugs in engineered EV.

Although EVs can be secreted by various types of cells and have unique properties, such as having specific ligands, further targeting capabilities are needed for their application as therapeutic carriers. Engineering the EVs surface can be an effective way to address this issue. Currently, there are exogenous and endogenous methods for surface modification of EVs to enhance targeting specificity. As shown in Section 2.3, EV surface membranes have been successfully modified with molecules such as peptides, antibodies, and aptamers for therapeutic targeting purposes in the body (Figure 4).

### 4.1. Peptide

Recently, many functional peptides have been established by the progression of peptide design technology, which is promising in the generation of diagnostic and therapeutic agents [259–261]. The development of EVs with peptide ligands is crucial for the delivery of drugs to specific organs and tissues. The surface engineering of peptides allows for the incorporation of their functionality on the EV, as the amino acid sequence can be designed both to regulate the physicochemical properties of cell to EVs surface interaction and for targeting a specific receptor on the target cell surface. The diversity of amino acid combinations can be easily produced, and the sequence design adjusts hydrophobicity and ionization, both of which have a significant effect on the interaction between cells and EVs surface *in vitro* and *in vivo*. Surface presentation of plasmids on EVs can be achieved by plasmid expression of targeting peptides fused to EV transmembrane proteins of EVs, such as Lamp2b, C1C2, and PDGFR. For example, Kim et al. achieved targeting by fusing Lamp 2b with transferrin receptor binding protein T7 to achieve targeting on glioblastoma cells that overexpress transferrin on their surfaces [262]. The researchers loaded the fusion peptide endogenously by transfection, and then as a therapeutic, loaded antisense miRNA-21 by electroporation [262]. After intravenous injections of the T7 presenting antisense miRNA-21 loaded EVs to the mice, the researchers noted a decrease in brain tumor size 5 days post injection [262]. In contrast, Jia et al. used click chemistry to modify the EV surface with RGE [129]. This modification was done after loading the EVs with curcumin and SPION for therapeutic and imaging purposes respectively [129]. Hence, peptide surface engineering of EVs shows promise for their use in clinical therapeutics.

### 4.2. Antibody

The use of monoclonal antibodies for cancer therapy has been of great interest and development [263–265]. Antibodies act to obtain specific targeting of drugs in the form of antibody-drug conjugates (ADCs). They have sufficient antitumor activity to be approved and used in clinical practice. However, there are concerns that chemically binding antibodies to drugs may lead to drug inactivation and problems with drug release after the conjugate is taken up by cancer cells. Modified antibodies on the EVs membrane may circumvent these problems because the drug is encapsulated by the EV rather than the drug and the antibody is covalently bound. There is no set method for conjugating antibodies to EVs, but some methods used before include fusing C1C2 to an anti-EGFR antibody as seen in Koojimans et al [134]. Alternatively, Li et al. used a chemical approach that involved coating the EVs with antibodies by isolating them from A33 positive LIM1215 cells. The researchers then loaded the EVs with therapeutic doxorubicin and then combined them with SPIONS that were coupled with A33 antibodies [266]. The aim was that the A33 antibodies would bind to A33 positive EVs to achieve A33 targeting, as A33 is highly expressed in colorectal cancers. Pham et al. in contrast did not use a genetic or chemical approach to surface engineering antibodies on EVs, but rather an enzymatic method [267]. The researchers used Sortase A and OaAEP1, protein ligating enzymes, to conjugate EVs with anti-EGFR antibodies to achieve targeted delivery to EGFR positive lung cancer cells and mice. Therefore, antibody use in conjugation with EVs shows a lot of potential for targeted drug delivery.

### 4.3. Aptamer

Aptamers are single-stranded RNA or DNA molecules that bind specifically to target molecules by adopting a complex 3D structure. Target molecules of aptamers include a wide range of proteins, peptides, carbohydrates, lipids, and low molecular weight compounds. Due to their high binding affinity and specificity as well as antibodies, they have been used in various fields, such as therapeutics and diagnostics [268]. Aptamers are generated using a PCR-based *in vitro* selection strategy known as the systematic evolution of ligands by exponential enrichment (SELEX) method [269,270]. Aptamers have a shorter half-life in blood compared to antibodies, while antibodies are difficult to remove from the body, making it challenging to deal with any abnormalities that may occur after administration. The development of drugs to neutralize aptamers is also easier than for antibodies. Antibodies administered therapeutically are foreign to the body and often become antigens, resulting in the generation of anti-drug antibodies in the patient, which can limit treatment. Aptamers, however, are synthetic molecules that generally do not produce anti-drug antibodies and are considered to be safe. For example, Wan et al. used AS1411, a nucleolin targeting aptamer anchored to dendritic derived EVs and loaded with paclitaxel, a chemotherapeutic drug, to achieve targeted drug delivery *in vivo* and *in vitro* [135]. Similarly, Luo et al. conjugated a bone marrow stromal cell (BMSC) targeting aptamer to BMSC-derived EVs that allowed for the accumulation of the EVs in the bone of the mouse model, resulting in a targeted treatment option for osteoporosis and bone fractures as illustrated *in vitro* and *in vivo* [271]. Thus, aptamers have the potential to overcome some of the difficulties associated with using antibodies in targeted EV cargo delivery.

## 5. EV-mediated delivery of therapeutic drugs

EVs inherently have a role in intercellular communication by acting as transportation vehicles of nucleic acids and proteins. This ability of EVs can be used for the in vivo delivery of therapeutic agents such as siRNAs, miRNAs, other RNAs, proteins, and drugs (Figure 4). Each therapeutic agent has its own advantages and disadvantages in terms of loading, dosage, ease of use, efficiency, and possible resistance, which are discussed below.

### 5.1. siRNA

siRNAs are a type of mRNA silencing molecule, typically 21–23 nucleotides long, that functions within the RNA interfering (RNAi) pathway [272,273]. They recognize the target mRNA through complementary base pairing, which leads to the degradation of the mRNA and silencing of that gene. siRNAs have been found to have many targets in many disease pathways, creating the possibility of using them in therapeutics. Using siRNA solely as a therapeutic agent has some barriers, such as that free siRNA has trouble crossing biological membranes due to its negative charge, thus causing it to fail to enter target cells. Additionally, siRNAs have a short half-life in their naked form and can cause possible off-target effects [273–275]. Due to these downsides of using naked siRNAs as therapeutics, they are often packaged into carriers that allow for better penetration and delivery. EVs as a targeted delivery method for siRNAs is a popular therapeutic option [70,276–278]. There is no one set method of loading EVs with siRNAs, there are multiple options, such as ultrasonic [274], electroporation [279], and PEI [280]. Tao et al. studied the anticancer effects of bcl-2 siRNA coated EVs compared with bcl-2 siRNA coated lipofectamine 3000^TM^ in digestive system cancers [274]. In their transfection efficiency in cancer cells, inhibition of migration, and promotion of apoptosis studies, EVs coated with bcl-2 siRNA had higher delivery and apoptosis rates, and lower expression of migration related proteins compared to the lipofectamine delivery method *in vitro* and *in vivo* [274]. There are other forms of modifications outside of EVs that allow for the delivery of siRNAs as a therapeutic, like gold nanoparticles, stable nucleic acid-lipid particles (SNALPS), liposomes, but they come with their own set of problems with toxicity, charge, delivery complications [272].

### 5.2. miRNA

MicroRNAs (miRNAs) are small non-coding RNAs, typically 20–22 nucleotides long. Like siRNAs, they bind to mRNAs through complementary base pairing [281]. This binding leads to either the degradation of the mRNA or inhibition of the mRNA’s translation. miRNAs have roles in almost all biological functions due to their constant presence and regulation of key genes in maintaining homeostasis. Any alterations in certain miRNA levels can promote disease progression, typically seen in cancers. For example, O’Brien et al. noticed miR-379 expression was reduced in breast cancer tissues of patients, and others have reported decreased expression in hepatocellular carcinoma and osteosarcoma tissues [282–285]. They engineered mesenchymal stem cells to secrete miR-379 enriched EVs and noticed their therapeutic potential after administering them into mice [282]. Wang et al., alternatively, noticed low levels of miR-335 in human liver cells [286]. To test if miR-335 can be used against hepatocellular carcinoma progression, they endogenously loaded miR-335 into L×2 cells and isolated the loaded EVs [286]. The researchers tested these EVs in both cancer cell lines and HCC infected mice and found that EV loaded miR-335 inhibited cancer progression and invasion [286]. When naked miRNAs are administered, they are prone to early degradation and are limited by the accessibility of the administration site. There are many clinical studies utilizing miRNAs with liposomal, or locked nucleic acid (LNA) delivery systems, and while none are using EVs yet, clinical trials with siRNAs will pave the path for miRNA in EV therapeutic delivery systems [287]. As EVs are natural carriers of miRNAs, the delivery system has few faults, if any, outside of the difficulties associated with EV production.

### 5.3. Other RNAs and DNAs

Outside of siRNAs and miRNAs, other common genetic-based drugs used for EV-mediated therapeutic delivery include mRNAs, DNA, gRNA, shRNA, CRISPR/Cas9. Rather than indirectly affecting mRNA expression levels, direct mRNA delivery is straightforward and simple. An example of mRNAs delivery by EVs used as a therapeutic can be seen in Erkan et al [288]. They genetically engineered EVs to carry the mRNA of a suicide gene, cytosine deaminase, fused to an uracil phosphoribosyltransferase (UPRT) and injected it into glioblastoma tumor mice models [288]. They found that the mRNA carrying EVs suppressed tumor growth by 70% compared to the control [288]. These researchers also noted the inhibitory effect of the mRNA suicide gene against schwannoma [289]. Alternatively, mediated delivery of DNA by EVs can be seen in Morishita et al. who loaded biotinylated CpG DNA into EVs [290]. They presented the CpG DNA on the surface by first transfecting B16BL6 cells with a fusion of biotin binding protein streptavidin (SAV) and the EV surface protein lactadherin (LA) [290]. The SAV-LA presenting EVs was isolated and combined with biotinylated CpG DNA, which allowed for the binding to SAV and presentation of the DNA [290]. The mediated delivery of CRISPR/Cas9 can be seen with Kim et al. who through electroporation loaded cancer cell-derived EVs with CRISPR/Cas9 and PARP-1 sgRNA [100]. By turning on apoptotic pathways, the researchers found that these EVs inhibited cancer cell proliferation *in vivo* and in vitro, and cancer cell-derived EVs had better delivery and accumulation compared to epithelial-derived EVs [100]. Besides miRNA and siRNA, other nucleic acids like DNA and mRNA are also being investigated for their EV-mediated therapeutic potential.

### 5.4. Proteins

In addition to small molecules, proteins can also be loaded into EVs and used in therapeutic delivery. There are several types of proteins that are commonly used, such as enzymes, peptides, cytokines, and cytoskeletal and transmembrane proteins [291]. The main issue that comes with using proteins as therapeutics compared to other forms of RNA is loading. Loading of proteins into EVs can protect them from degradation and deactivation, and possibly enhance enzymatic activity as Haney et al. showed [201]. These researchers loaded catalase, an antioxidant, *ex-vivo* into EVs using multiple loading techniques, like sonication, freeze-thaw, and saponin [201]. They found that sonication and saponin had the highest loading efficiency and release of active catalase [201]. Besides directly loading proteins into EVs, another method involves changing the protein expression of the parent cell directly and isolating the resulting EVs produced. Huang et al. produced bone morphogenetic protein 2 (BMP2) loaded EVs by transducing MSC with lentiviruses that expressed BMP2 and isolating EVs from the resultant cell line [292]. The researchers, after confirming the expression of BMP2, tested bone regeneration in rat calvarial defect models. The BMP2 EVs promoted greater bone regeneration compared to the control [292]. There are cases where it might be better to directly load proteins, rather than miRNAs and siRNAs that indirectly affect protein expression.

### 5.5. Chemical drugs

As of late, nanoscale systems have been investigated for drug delivery to improve their therapeutic outcomes and reduce side effects associated with traditional administration methods [293]. Liposomes and polymetric nanoparticles are some of the more commonly used nanoparticle-based drug delivery systems. However, these delivery vehicles are not ideal due to their lack of stability, poor biocompatibility, increased toxicity, and low circulation time in the body [294]. Using EVs as drug delivery vehicles has the potential to overcome the issues of alternative delivery methods [291]. Engineered EVs could be loaded with chemical drugs to provide a new approach to tumor treatment, especially chemotherapy and photodynamic therapy (PDT). EVs have specifically shown promise in chemotherapeutic drug delivery for cancers, such as in the cases of the drugs doxorubicin, paclitaxel, methotrexate, and curcumin [295]. Tian et al. showed that dendritic cell-derived EVs with alpha V integrin-specific iRGD peptide were able to successfully deliver doxorubicin cargo loaded via electroporation to tumor cells [296]. Additionally, Kim et al. demonstrated that macrophage-derived EVs loaded with paclitaxel could be used for targeted delivery to lung cancer cells [136]. PDT has been used for a long time for anti-tumor therapy as well as chemotherapy [297,298]. PDT is a treatment that combines light energy with special drugs, called photosensitizers, to kill tumor cells after light activation. Using this method, Foslip, a formulation of liposomes mixed with a photosensitizer (mTHPC), has limited clinical applications due to its low accumulation at tumor sites because it is prematurely eliminated from the bloodstream [299]. Pinto et al. have shown that MSC-EVs encapsulating mTHPC improve tumor selectivity and activates the tumor immune environment compared to liposome-complexes [300]. Overall, EVs show a lot of promise as a drug delivery method.

## 6. Clinical trial of engineered EVs

Clinical trials on EV-based therapies have been growing rapidly. In April 2022, 53 clinical studies for therapeutics involving engineered EVs (Table 3) and natural EVs have been registered on the www.Clinical Trial.gov database. Currently, there is one phase I clinical trial (NCT03608631) ongoing from the MD Anderson Cancer Center that uses mesenchymal stromal cells derived EVs and loads them with KRAS G12D siRNA to treat patients with pancreatic cancer. The mRNA vaccine was quickly approved as a prevention vaccine for COVID-19 (BNT162b2; Pfizer and BioNTech) due to previous achieved findings, which has triggered research using mRNA. Familial hypercholesterolemia (FH) is caused by mutations in the gene encoding the low-density lipoprotein (LDL) receptor, which is responsible for removing LDL from the bloodstream. A *Ldlr* mRNA therapeutic drug loaded into EVs has successfully reduced the number and size of atherosclerotic plaques and inflammation in *Ldlr*-deficient mice and is enrolled in a clinical trial (NCT05043181) as a new treatment for patients with FH disease [301]. There have also been several clinical trials on the therapeutic use of drug-loaded EVs. Ma et al. performed a clinical trial (NCT01854866) that showed that microparticles were able to successfully deliver anti-cancer drugs to tumor sites in lung cancer patients [302]. Guo et al. carried out a clinical trial (NCT02657460) that showed tumor-cell derived microparticles were able to successfully package and deliver methotrexate to lung cancer cells, resulting in a decrease in tumor size [303]. A third clinical trial (NCT01294072) on EV-based drug delivery is currently being carried out to investigate the use of plant-derived EVs packaged with curcumin as a treatment for colon cancer [304]. In addition, COVID-19 is known to cause excessive production of early response proinflammatory cytokines (TNF, IL-6, IL-1beta), which is called cytokine storm syndrome, and continued excessive production increases the risk of increased vascular permeability, multiple organ failure, and ultimately death [305]. Therefore, since CD24 has a role in regulating the homeostatic proliferation of T cells, CD24-overexpressing EVs may be able to suppress cytokine storms, and their safety and efficacy have been registered for evaluation (NCT04969172, NCT04747574, and NCT04902183). Codiak Biosciences has successfully demonstrated the application of using HEK293 cells to design EVs to carry various types of therapeutic molecules and deliver them to specific target cells. Two types of engineered EVs in development are currently enrolled in Phase I and II clinical trials to validate the safety and efficacy of the EVs against Advanced Solid Tumor (NCT04592484) and Cutaneous T-cell Lymphoma (NCT05156229) for their clinical application. The first exoSTING is a STimulator of InterferoN Genes (STING) agonist loaded on the lumen of the EVs, which causes the exosomal protein prostaglandin F2 receptor negative regulator [306,307]. Overall, engineered EVs can compensate for the lack of naïve EVs by effective cargo engineering and specific surface engineering, and the number of clinical studies enrolled is expected to increase in the future. There are still many unclear things about EVs, and further knowledge needs to be acquired.

**Table 3.** List of currently registered clinical trial of engineered EV from the www.Clinical Trial.Gov database.

Sponsor	Therapeutic Agent	Delivery System	Disease/conditions	Clinical trail
Athens Medical Society	Exosomes overexpressing CD24	Inhalation	Covid19	Phase II (NCT04902183), Recruiting (Jul. 2021)
Codiak BioSciences	CDK-002 (exoSTING)	Intratumorally	Advanced Solid Tumor	Phase I/II(NCT04592484), Recruiting (Dec. 2021)
Codiak BioSciences	CDK-003 (exoIL-12)	Intratumorally	Cutaneous T-cell Lymphoma (CTCL)	Phase I(NCT05156229), Recruiting (Dec. 2021)
Eli Sprecher, MD	Exosomes overexpressing CD24 from human embryonic kidney *T*-REx™-293	Inhalation	SARS-CoV-2	Phase II (NCT04969172), Active, not recruiting (Jul. 2021)
Huazhong University of Science and Technology	Tumor cell-derived microparticles packaging chemotherapeutic drugs	Locally administration	Malignant Pleural Effusion, Malignant Ascites	Phase II(NCT01854866), Unknown (Oct. 2013)
Hui ting Xu, MD	Autologous Erythrocytes Derived MPs Packaging MTX		Malignant Ascites	Phase I/II(NCT03230708), Unknown (Jul. 2017)
M.D. Anderson Cancer Center	Mesenchymal Stromal Cells-derived Exosomes with KRAS G12D siRNA	Intravenous administration	Pancreatic cancer.	Phase I(NCT03608631), Recruiting (Apr. 2021)
Peking Union Medical College	Microparticles packaging methotrexate (MPs-MTX)	Intrapleural administration	Lung Cancer Breast Cancer Malignant Pleural Effusion	Not applicable (NCT04131231), Unknown (Oct. 2019)
Tang-Du Hospital	Low Density Lipoprotein Receptor mRNA Exosomes (MSC)	Intravenous/peritoneal administration	Familial Hypercholesterolemia	Phase I(NCT05043181), Not yet recruiting (Sep. 2021)
Tel-Aviv Sourasky Medical Center	EXO-CD24	Inhalation	SARS-CoV-2	Phase I (NCT04747574), Recruiting (Mar. 2021)
University of Louisville	Plant EV curcumin	Oral administration	Colon Cancer	Phase I (NCT01294072), Recruiting (Jun. 2021)
University of Louisville	Plant EV curcumin		Irritable Bowel Disease	Not applicable (NCT04879810), Recruiting (Feb. 2022)
Wuhan Union Hospital	Tumor Cell-derived Microparticles Packaging Chemotherapeutic methotrexate		Malignant Pleural Effusion	Phase II(NCT02657460), Unknown (Feb.2019)

## 7. Conclusions

EVs have been identified as essential mediators in intercellular communication, transferring various bioactive molecules between cells such as lipids, proteins, RNAs, and DNAs. EV-mediated cellular communication plays an essential role in pathophysiological and physiological processes. Thus, these properties make them an attractive candidate for disease biomarkers and therapeutic medicine. In therapeutics, EVs have attracted great attention due to their low immunogenicity, inherent targeting ability, and capacity to protect incorporated bioactive molecules. Although naïve EVs have therapeutic effects in preclinical studies for a variety of diseases, they have not been approved for use as a drug currently. Therefore, the engineering of EVs, such as by loading them with exogenous therapeutics or modifying EV membranes with tissue or site-specific targeting molecules, is being advanced as a strategy to overcome the limitations with targeting and loading. However, engineered EV use is still in the early research stage and is currently being optimized for clinical application. In addition, to make EVs available as a therapeutic, we need to study methods involving their separation, storage, quality, and large-scale production. Despite the challenges, growing EV based research has shown great potential in many preclinical and clinical studies. Engineering EVs show promise to advance the next generation of EV-based therapies.
